# Inverse kinematics for cooperative mobile manipulators based on self-adaptive differential evolution

**DOI:** 10.7717/peerj-cs.419

**Published:** 2021-03-08

**Authors:** Jesus Hernandez-Barragan, Carlos Lopez-Franco, Nancy Arana-Daniel, Alma Y. Alanis

**Affiliations:** Department of Computer Science, University of Guadalajara, Guadalajara, Jalisco, Mexico

**Keywords:** Inverse kinematics, Cooperative systems, Mobile manipulators, Differential evolution

## Abstract

This article presents an approach to solve the inverse kinematics of cooperative mobile manipulators for coordinate manipulation tasks. A self-adaptive differential evolution algorithm is used to solve the inverse kinematics as a global constrained optimization problem. A kinematics model of the cooperative mobile manipulators system is proposed, considering a system with two omnidirectional platform manipulators with n DOF. An objective function is formulated based on the forward kinematics equations. Consequently, the proposed approach does not suffer from singularities because it does not require the inversion of any Jacobian matrix. The design of the objective function also contains penalty functions to handle the joint limits constraints. Simulation experiments are performed to test the proposed approach for solving coordinate path tracking tasks. The solutions of the inverse kinematics show precise and accurate results. The experimental setup considers two mobile manipulators based on the KUKA Youbot system to demonstrate the applicability of the proposed approach.

## Introduction

Dual-arm systems are commonly used to perform complex cooperative tasks such as human-like tasks in domestic and industrial environments (Yang et al., 2019). However, the cooperative tasks for dual-arm systems are limited by the workspace provided by the robot manipulators. Mobile platforms can be used to extend the workspace of the manipulators. For this reason, a cooperative system conformed by two mobile manipulators increases the capabilities with greater accessibility to perform a cooperative task. Dual-arm manipulation can be defined as physical interaction with an object, exerting forces to move or reshape it using two robot manipulators (Smith et al., 2012). There are two main types of robotic manipulation, non-coordinated manipulation, and coordinated manipulation. In non-coordinate manipulation, manipulators perform different tasks independently. In this case, the motion of each manipulator can be analyzed separately. In coordinated manipulation, manipulators are physically interacting with each other. In this case, the relative motion has to be adequately controlled to perform a task. These definitions of cooperative manipulation are not limited to dual-arm systems. Indeed, such concepts have been applied to different cooperative robot systems (Korayem & Dehkordi, 2019a). This work introduces a model description of a cooperative mobile manipulators system for coordinate manipulation tasks.

The relative Jacobian matrix is commonly used to solve the coordinate manipulation of dual-arm systems (Lewis, 1996; Jamisola & Roberts, 2015). This method considers the dual-arm system as a single redundant manipulator. Therefore, control algorithms for a single manipulator can be used to control dual-arm systems modeled with the relative Jacobian matrix. The relative Jacobian matrix is extended with the Jacobian null space projection for cooperative tasks of redundant manipulators (Jamisola & Roberts, 2015; Freddi et al., 2016).

Kinematic and dynamic control strategies have been presented to deal with cooperative manipulation. Coordinated cooperative controllers for trajectory tracking of a dual-arm system mounted on an omnidirectional platform is presented in Molina & Ortiz (2018) and Ortiz et al. (2018). The control strategy is based on the kinematic modeling of the entire system, and it allows to transport a common object. In Mora-Aguilar et al. (2018), the authors describe a centralized cooperative control algorithm for two heterogeneous mobile manipulators. The cooperative system is conformed by a mobile differential platform manipulator and an omnidirectional platform manipulator. This control strategy also considers the kinematic model for the two heterogeneous mobile manipulators. A cooperative transportation control strategy for multiple mobile manipulators is proposed in Chen & Kai (2018). This work deals with the problem of distributed control for an object tracking with energy and manipulability optimization. The control strategy is based on the kinematics of redundant robots. In Wu, He & Liu (2016), the authors present a leader-follower structure for cooperative manipulation of two mobile manipulators. The compliance feature is achieved with a classic PID controller, which considers the system dynamic equations. In Pajak (2019), the authors propose a real-time trajectory generation for two cooperating mobile manipulators moving a common rigid object. This proposal introduces a leader-follower control scheme based on the kinematics and dynamics of the system. Moreover, control limitations and collision avoidance are considered. In Korayem & Dehkordi (2019a), the authors present the dynamics equation of closed kinematics cooperative mobile manipulator. The motion control is achieved based on the kinematic chain and applying it in the dynamic model. The dynamic model comprising *M* manipulators are fixed in a nonholonomic mobile platform. The same authors used these principle concepts to develop a dynamic model for multiple mobile manipulator systems (Korayem & Dehkordi, 2019b). A fully distributed cooperative scheme for a networked mobile manipulator is proposed in Ren, Sosnowski & Hirche (2020). Distributed adaptive controllers are used to achieve motion synchronization, which deals with kinematics and dynamics uncertainties. Manipulation experiments with three mobile manipulators confirm the applicability in the distributed cooperation task.

Motion and path planning for cooperative manipulation have also been presented. In Tallamraju et al. (2019), a kinematic motion planning algorithm for cooperative manipulation is proposed. A convex decentralized model-predictive controller is formulated to plan collision-free trajectories for the robot formation. The problem of path planning for spatial payload transportation for mobile manipulators is addressed in Tallamraju, Sripada & Shah (2019). This approach uses non-linear multi-objective optimization to compute optimal paths for the robot formations. Moreover, rapidly-exploring random trees are used to find a kinematically feasible and collision-free trajectory.

The mentioned kinematics and dynamics control strategies use the information of a Jacobian matrix to map the end-effector velocities to the manipulator joint velocities. The joint velocities are used to control the motion of the mobile manipulator, and the end-effector velocities are commonly computed by an error between a desired and a current end-effector positions. The drawback of these methods is the singularities that occur when the Jacobian matrices become rank deficient (Hu, Huang & Yang, 2015). Singularities represent joint configurations where the mobility of the manipulator is reduced, infinite solutions for the inverse kinematics may exist, small velocities of the end-effector may cause large velocities for the joint configuration (Siciliano et al., 2008). To overcome this inconvenience, this work introduces a kinematic model of cooperative mobile manipulators systems, which is based only on forward kinematics equations. The solutions of the inverse kinematics are obtained based on a global optimization problem, where it is not required the inclusion of any Jacobian matrix.

In a mobile manipulator system, the combination of the degrees of freedom (DOF) of the mobile platform and the manipulator becomes the system into a redundant robot. Consequently, a cooperative mobile manipulator system is considered redundant. Redundancy occurs when the degree of motion is higher than the number of necessary variables to perform a task (Freddi et al., 2016). The inverse kinematics for redundant robots becomes difficult to solve because redundancy admits several joint configurations to reach the same end-effector pose. For this reason, meta-heuristic algorithms are commonly applied to solve the inverse kinematics problem. A trajectory planning of robot manipulators is proposed in Savsani, Jhala & Savsani (2016). The authors included a comparative study among the artificial bee colony (ABC), biogeography-based optimization (BBO), the gravitational search algorithm (GSA), cuckoo search algorithm (CS), firefly algorithm (FA), bat algorithm (BA), and teaching-learning-based optimization (TLBO) algorithms. The TLBO, ABC, and CS algorithms showed a significant improvement over the others.

The inverse kinematics solutions for robot manipulators is presented in Ayyldz & Çetinkaya (2016). This work included a comparative study among the genetic algorithm (GA), particle swarm optimization (PSO), quantum particle swarm optimization (QPSO), and GSA algorithms. The comparative results showed that QPSO is more useful to solve the inverse kinematics than the others. In Ziwu, Chunguang & Lining (2016), a trajectory optimization method for a humanoid manipulator based on differential evolution (DE) is proposed. The DE algorithms minimize the maximum acceleration of the trajectory. PSO algorithm has been used to solve the inverse kinematics for high-DOF inverse kinematics (Collinsm & Shen, 2017). The authors compared the performance of the Constriction factor PSO (CFPSO) and Bare Bones PSO (BB-PSO). The results reported that CFPSO is more convenient to solve the inverse kinematics for high-DOF robots. In El-Sherbiny, Elhosseini & Haikal (2018), a new variant of ABC is introduced to solve the inverse kinematics of robot manipulators, which is called KABC. The reported results prove that the KABC algorithm performed better than the classical ABC. A PSO algorithm was used to solve the coordinated trajectory planning of dual-arm space robot (Wang et al., 2018). In this case, the PSO version uses dynamic weighting factors, and it can handle constraints.

In Kumar et al. (2019), the ABC and Grey Wolf Optimization (GWO) algorithms are considered to solve the inverse kinematics for arm manipulators. The ABC algorithm performed better than GWO with a better minimum error. The path planning optimization of robotic manipulators has been addressed in Šegota et al. (2020). The authors included a comparison among the GA, Simulating Annealing (SA), and DE algorithms, where GA proves to be better than the other algorithms. The Flower Pollination Algorithm (FPA) is another promising meta-heuristic algorithm (Yang, 2012). It has been recently applied successfully to solved mobile robots applications (Low, Ong & Cheah, 2019; Mishra & Deb, 2020). Based on the studies mentioned above, the DE algorithm has shown to be adequate to solve inverse kinematics problems. However, in Fan, Xie & Zhou (2019) a new variant of DE is proposed to improve the local exploitation and global exploration, with convergence accuracy and fast converge rate. This version is called self-adaptive mutation differential evolution constrained optimization (SDE), and it has been used to determine the optimum path generation of a rock-drilling manipulator with nine DOF based on the SDE algorithm. The SDE algorithm solves the inverse kinematics for a single manipulator system. In this work, it is proposed to solve the inverse kinematics of cooperative mobile manipulator system based on the SDE algorithm. This proposed approach introduces the kinematics model for the cooperative system which considers two omnidirectional platform mobile manipulators. Moreover, a comparative study is included to compare the performance of SDE against CFPSO, FPA and KABC.

The contributions of this paper are summarized as follows: The inverse kinematics of cooperative mobile manipulators is solved as a global constrained optimization problem. A kinematic model for the cooperative mobile manipulator system is proposed and an objective function is formulated based on the forward kinematics equations. The proposed approach avoids singularity configurations since it does not require the use of any Jacobian matrix. The formulation of the objective function includes the use of the penalty function to handle constraints. The constraints represent the join limits of the mobile manipulators, which is crucial for real-world implementations. Finally, the considered cooperative mobile manipulators are conformed by two omnidirectional platform manipulators with *n* DOF.

This article is organized as follows: The “Cooperative Mobile Manipulator System” section provides the kinematic model of the considered cooperative system which is conformed by two omnidirectional platform manipulators. In the section “Description of the Proposed Approach”, the objective function is formulated, the description of the SDE algorithm is provided, and the inverse kinematics algorithm for cooperative mobile manipulators is presented. The “Experimental Results” section presents the results of the proposed approach solving cooperative path tracking tasks. The comparison study is also included in this section. Finally, the conclusion is given in the “Conclusions” section.

### Cooperative mobile manipulators system

This work introduces an approach to solve the inverse kinematics for cooperative mobile manipulators system. The proposed method is based on the forward kinematics equations. In this section, kinematics concepts and important details are presented. First, a brief description about the Denavit-Hartenberg model for the kinematics equations of robotic manipulators is presented. Then, a kinematic model for a mobile manipulator is presented, where a manipulator of *n* total degrees of freedom (DOF) attached to an omnidirectional mobile platform of three DOF is considered. Finally, the forward kinematics equations for a cooperative mobile manipulators model are provided.

### Manipulator kinematics

Robotic manipulators are conformed by a series of joints connected by links that represent a kinematic chain. In a manipulator with an open kinematic chain, the total DOF are defined by the total number of joints (Spong & Vidyasagar, 2008; Siciliano et al., 2008). A joint variable **q***_m_* is defined as follows

(1) }{}{\bf q}\mathop {}\nolimits_m = {\left[ {\matrix{ {{q_1}} & {{q_2}} & {{q_3}} & \cdots & {{q_n}} \cr } } \right]^T}

where each joint value *q*_*j*_ with *j* = 1,2,3,…,*n* represents an articulation, and *n* is the total DOF.

Commonly, revolute or prismatic joints are used to represent an articulation. The joint value *q*_*j*_ is an angle of rotation for revolute joints. Otherwise, the joint value *q*_*j*_ is a displacement for prismatic joints. In other words

(2) }{}{q_j} = \left\{ {\matrix{ {{{\rm{\theta}} _j}} {{\bf if} \;\rm joint}\; j\; \text{is\;revolute} \cr {{d_j}} {{\bf if} \;\rm joint}\; j\; \text{is\;prismatic} \cr } } \right.

**Figure 1:**
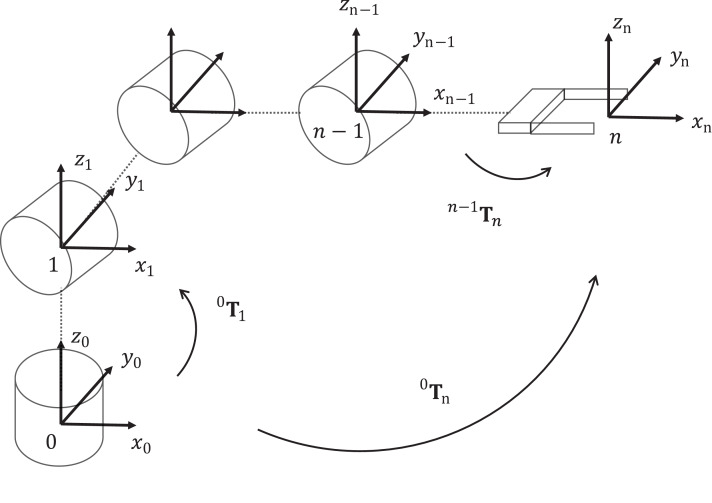
Kinematic chain of robotic manipulators. The homogeneous matrix }{}^{j - 1}{\bf T}_j for *j* ∈ 1,2,3,…,*n* represents the coordinate frame of the link *j*

The manipulator kinematics model can be obtained based on its kinematics chain, see Fig. 1. Each link *j* is represented by a homogeneous matrix ^*j*−1^**T**_*j*_ that transforms the frame attached to the link *j* − 1 into the frame link *j*. The transformation ^0^**T**_*n*_ represents a homogeneous matrix that contains the position and orientation of the end-effector. The kinematics model can be described using the Denavit–Hartenberg (DH) convention, where the homogeneous matrix ^*j*−1^**T**_*j*_ is expressed as

(3) }{}^{j - 1}{{\bf T}_j} = \left[ {\matrix{ {c{{\rm{\theta}} _j}} { - s{{\rm{\theta}} _j}c{{\rm{\alpha}} _j}} {s{{\rm{\theta}} _j}s{{\rm{\alpha}} _j}} {{a_j}c{{\rm{\theta}} _j}} \cr {s{{\rm{\theta}} _j}} {c{{\rm{\theta}} _j}c{{\rm{\alpha}} _j}} { - c{{\rm{\theta}} _j}s{{\rm{\alpha}} _j}} {{a_j}s{{\rm{\theta}} _j}} \cr 0 {s{{\rm{\alpha}} _j}} {c{{\rm{\alpha}} _j}} {{d_j}} \cr 0 0 0 1 \cr } } \right]

where θ_*j*_ is a joint angle, *a*_*j*_ is a link length, *d*_*j*_ is a link offset and α_*j*_ is a link twist. For brevity, the sin and cos operations are represented with the letters *s* and *c*, respectably. When a manipulator has a revolute joint, then the parameter θ_*j*_ becomes the joint variable and the other parameters remain constant. When the manipulator has a prismatic joint, the parameter *d*_*j*_ becomes now the joint variable and the rest of the parameters remain constant.

The forward kinematics consists in computing the end-effector pose ^0^**T**_*n*_ given the joint variable }{}{\bf q}\mathop {}\nolimits_m. The forward kinematics can be computed as

(4) \matrix{ {^0{{\bf T}_n}({\bf q}\mathop {}\nolimits_m )} \hfill {{ = ^0}{{\bf T}_1}({q_1}){\,^1}{{\bf T}_2}({q_2}){\,^2}{{\bf T}_3}({q_3})\,\, \cdots \,{\,^{n - 1}}{{\bf T}_n}({q_n})} \hfill \cr {} \hfill { = \prod\limits_{j = 1}^n {\;^{j - 1}}{{\bf T}_j}({q_j})} \hfill \cr }

where the matrix ^0^**T**_*n*_ is expressed as

(5) }{}^0{{\bf T}_n}({{\bf q}_{\mathop {}\nolimits_m }}) = \left[ {\matrix{ {{r_{11}}} & {{r_{12}}} & {{r_{13}}} & {{t_x}} \cr {{r_{21}}} & {{r_{22}}} & {{r_{23}}} & {{t_y}} \cr {{r_{31}}} & {{r_{32}}} & {{r_{33}}} & {{t_z}} \cr 0 & 0 & 0 & 1 \cr } } \right] = \left[ {\matrix{ {\bf R} & {\bf t} \cr {\bf 0} & 1 \cr } } \right]

where the orientation of the end-effector is represented by the matrix **R**, and its Cartesian position is given by the vector }{}{\bf t}. More detailed information manipulator kinematics can be found in Craig (2005), Spong & Vidyasagar (2008) and Siciliano et al. (2008).

### Mobile manipulator kinematics

Mobile manipulators are conformed by one or more manipulators attached to a mobile platform. Conventional mobile robots such as unicycle, differential drive, and car-like mobile robots are used to increase the manipulator workspace. However, these platforms have limited movement capabilities due to their nonholonomic kinematics constraints (Li et al., 2016). Nonholonomic constraints limit some driving directions of a mobile robot. Conventional mobile robots are usually subjected to nonholonomic constraints, which means that not all driving directions are possible. There are also many possible solutions to arrive to the desired pose. The differential, tricycle, and car-like robots can move forward, backward, or rotate to change their orientation, but they cannot move in the lateral direction. In contrast, omnidirectional mobile platforms improve the movement capabilities, which allow them simultaneously to move towards any position and reach any desired orientation (Kundu et al., 2017; Wu et al., 2017). The omnidirectional mobile platforms have no limitation in their velocity space so all directions of motion in the state space are possible. This section introduces a kinematic model of a mobile manipulator conformed by a robotic manipulator of *n* DOF attached to an omnidirectional mobile platform.

**Figure 2:**
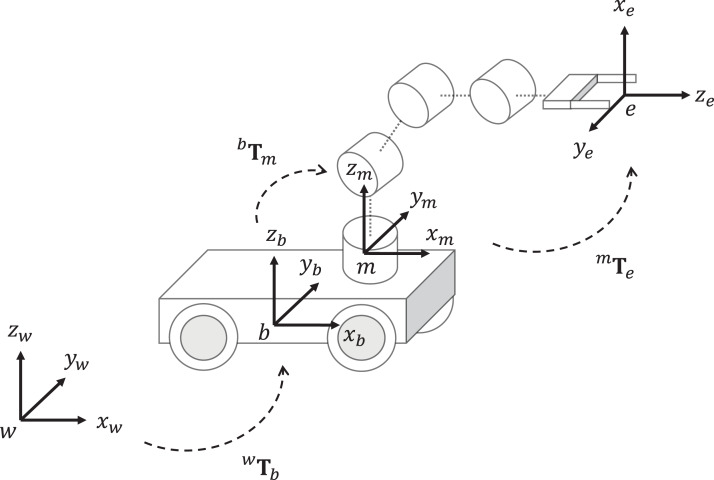
Kinematic chain of a mobile manipulators. The transformation *^w^***T***_b_* is the homogeneous matrix from the world frame *w* to the mobile platform base frame *b*, *^b^***T***_m_* is the homogeneous matrix from *b* to the manipulator base frame *m*, ^*m*^**T**_*e*_ is the homogeneous matrix from m to the end-effector frame *e*.

The kinematic chain of mobile manipulators is described in Fig. 2. The homogeneous matrix ^*w*^**T**_*b*_ defines the position and orientation of the mobile platform. The transformation ^*b*^**T**_*m*_ is a constant homogeneous matrix between the mobile platform frame and the manipulator base. The matrix ^*m*^**T**_*e*_ can be computed based on the DH model of the manipulator.

Considering an omnidirectional mobile platform, the pose of the robot with respect to the world frame *w* is given by three DOF, which are the positions *x*_*b*_ and *y*_*b*_, and the orientation θ_*b*_. Then, the matrix ^*w*^**T**_*b*_ can be defined as

(6) }{}^w{{\bf T}_b} = \left[ {\matrix{ {\cos ({{\rm{\theta}} _b})} & { - \sin ({{\rm{\theta}} _b})} & 0 & {{x_b}} \cr {\sin ({{\rm{\theta}} _b})} & {\cos ({{\rm{\theta}} _b})} & 0 & {{y_b}} \cr 0 & 0 & 1 & 0 \cr 0 & 0 & 0 & 1 \cr } } \right]

The matrix ^*b*^**T**_*m*_ is constant and it adjusts the distance from the mobile platform base frame *b* to the manipulator base frame *m*. The values *d*_*x*_, *d*_*y*_ and *d*_*z*_ are used to adjust the distance in the direction of the *x*-axis, *y*-axis and *z*-axis, respectively. If the frame orientation is not needed to adjust, then the matrix ^*b*^**T**_*m*_ can be described by

(7) }{}^b{{\bf T}_m} = \left[ {\matrix{ 1 & 0 & 0 & {{d_x}} \cr 0 & 1 & 0 & {{d_y}} \cr 0 & 0 & 1 & {{d_z}} \cr 0 & 0 & 0 & 1 \cr } } \right]

Let us consider a joint variable }{}{\bf q} to represent the platform configuration \mathop {\bf q}\nolimits_b = {\left[ {\matrix{ {{x_b}} {{y_b}} {{{\rm{\theta}} _b}} \cr } } \right]^T} and the manipulator configuration }{}{\bf q}\mathop {}\nolimits_m = {\left[ {\matrix{ {{q_1}} & {{q_2}} & {{q_3}} & \cdots & {{q_n}} \cr } } \right]^T}. The joint variable for the mobile manipulator is given by }{}{\bf q} = {\left[ {\matrix{ {{\bf q}{{\mathop {}\nolimits_b }^T}} & {{\bf q}{{\mathop {}\nolimits_m }^T}} \cr } } \right]^T}.

Given the joint variable }{}{\bf q}, the computation of }{}^w{{\bf T}_e}\left( {\bf q} \right) which is the forward kinematics of the mobile manipulator can be obtained as

(8) }{}^w{{\bf T}_e}\left( {\bf q} \right){ = ^w}{{\bf T}_b}\left( {{\bf q}\mathop {}\nolimits_b } \right){\,^b}{{\bf T}_m}{\,^m}{{\bf T}_e}\left( {{\bf q}\mathop {}\nolimits_m } \right)

where }{}^w{{\bf T}_e}\left( {\bf q} \right) represents the end-effector pose with respect to the world frame *w*.

### Cooperative mobile manipulators model

In a cooperative mobile manipulators system, the mobile manipulators are physically interacting with each other, which means that their relative motions have to be adequately controlled to perform a task (Smith et al., 2012). This section introduces the kinematic model of a cooperative mobile manipulator system with omnidirectional mobile platforms.

**Figure 3:**
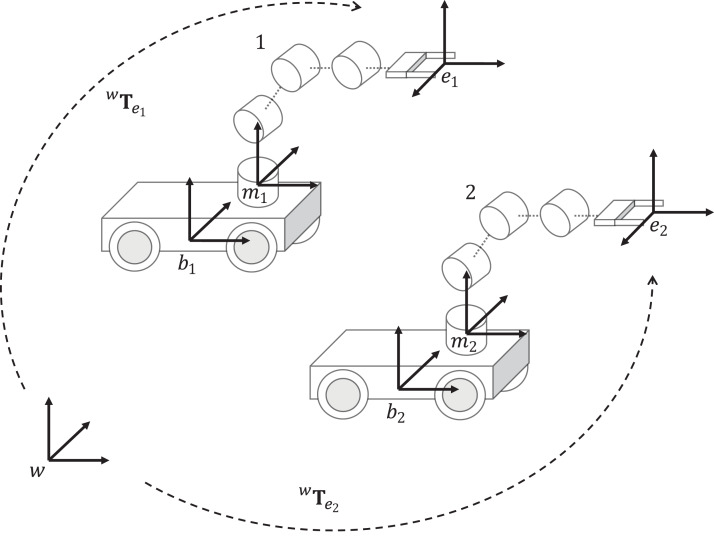
Cooperative mobile manipulator model. This model considers two mobile manipulators, where }{}^w{\bf T}_{{e}_1} represents the end-effector pose of mobile manipulator 1, and }{}^w{\bf T}_{{e}_2} represents the end-effector pose of mobile manipulator 2, both matrices are given respect to the world frame w.

The considered cooperative system is illustrated in Fig. 3. The homogeneous matrices }{}^w{{\bf T}_{{e_1}}} and }{}^w{{\bf T}_{{e_2}}} represent the kinematic model of the mobile manipulator 1 and 2, respectively. Let define two joint variables }{}{{\bf q}_1} and }{}{{\bf q}_2} to represent the configuration of mobile manipulator 1 and 2, respectively. The forward kinematics for each mobile manipulator can be computed as }{}^w{{\bf T}_{{e_1}}}\left( {{{\bf q}_1}} \right) and }{}^w{{\bf T}_{{e_2}}}\left( {{{\bf q}_2}} \right) using Eq. (8). Both matrices are defined as

(9) }{}^w{{\bf T}_{{e_1}}}({{\bf q}_1}) = \left[ {\matrix{ {{{\bf R}_{{e_1}}}} {{{\bf t}_{{e_1}}}} \cr {\bf 0} 1 \cr } } \right]

(10) }{}^w{{\bf T}_{{e_2}}}({{\bf q}_2}) = \left[ {\matrix{ {{{\bf R}_{{e_2}}}} & {{{\bf t}_{{e_2}}}} \cr {\bf 0} & 1 \cr } } \right]

where }{}{{\bf R}_{{e_1}}} and }{}{{\bf t}_{{e_1}}} represent the orientation and position of the end-effector of mobile manipulator 1. Similarly, }{}{{\bf R}_{{e_2}}} and }{}{{\bf t}_{{e_2}}} represent the orientation and position of the end-effector of mobile manipulator 2.

## Description of the proposed approach

This work introduces an approach to solve the inverse kinematics for cooperative mobile manipulators as a global constrained optimization. This section provides a detailed formulation of an objective function to solve the inverse kinematics, which includes penalty functions to handle constraints. The objective function is based on the forward kinematics equation of the cooperative system. It is proposed to use the self-adaptive differential evolution to solve the optimization problem. This section also provides a description of this variant of DE. Finally, a cooperative path tracking algorithm is presented based on the proposed inverse kinematics method.

### Objective function formulation

Let consider the cooperative mobile manipulator system of Fig. 3. Mobile manipulator 1 is considered to have the role of master. Then, the end-effector position of frame *e*_1_ is expressed with respect to the end-effector position of frame *e*_2_ using a vector }{}{{\bf t}_r}. The vector }{}{{\bf t}_r} defines the relative position between the end-effector position of mobile manipulators 1 and 2. In order to solve the inverse kinematics of the cooperative system, a desired position of the end-effector for the mobile manipulator with the role master must be provided. Then, the desired position for mobile manipulator 1 is defined as }{}{\bf t}_{{e_1}}^*. The desired position for mobile manipulator 2 is computed based on the definition of }{}{{\bf t}_r} as follows

(11) }{}{\bf t}_{{e_2}}^* = {\bf t}_{{e_1}}^* + {{\bf t}_r}

Let start by defining the actual joint configuration }{}{\bf x} of the whole cooperative system. This vector is represented as }{}{\bf x} = {\left[ {\matrix{ {{\bf q}_1^T} {{\bf q}_2^T} \cr } } \right]^T} with }{}{\bf x} \in {{\rm {\open R}}^{{n_1} + {n_2}}}, where *n*_1_ and *n*_2_ are the total DOF of manipulator 1 and manipulator 2, respectively. Using the actual joint configuration }{}{\bf x}, the end-effector pose for both mobile manipulators can be computed with the forward kinematics of mobile manipulators defined in Eqs. (9) and (10). Then, the positions }{}{{\bf t}_{{e_1}}} and }{}{{\bf t}_{{e_2}}} can be used to define position errors for both mobile manipulators.

The error between a desired position }{}{\bf t}_{{e_1}}^* and the actual position }{}{{\bf t}_{{e_1}}} for the end-effector of mobile manipulator 1 can be computed as

(12) }{}{t_{{1_{\rm error}}}} = \left\| {{\bf t}_{{e_1}}^* - {{\bf t}_{{e_1}}}} \right\|

The error between a desired position }{}{\bf t}_{{e_2}}^* and the actual position }{}{{\bf t}_{{e_2}}} for the end-effector of mobile manipulator 1 can be calculated as

(13) }{}{t_{{2_{\rm error}}}} = \left\| {{\bf t}_{{e_2}}^* - {{\bf t}_{{e_2}}}} \right\|

where }{}{\bf t}_{{e_2}}^* is defined in Eq. (11).

To minimize the cooperative manipulation motion, an error between the actual joint configuration }{}{\bf x} and an initial joint configuration }{}{{\bf x}_0} is defined. The vector }{}{{\bf x}_0} can also represent a previous joint configuration. This error is defined as

(14) }{}{x_{\rm error}} = \left\| {{\bf x} - {{\bf x}_0}} \right\|

Then, the formulation of an objective function *f* which consider the position errors }{}t_{1_{\rm error}} and }{}t_{2_{\rm error}}, and the error for the joint motion *x*_error_ is defined as

(15) }{}f = {\rm{\alpha}} \,\left( {{t_{{1_{\rm error}}}} + {t_{{2_{\rm error}}}}} \right) + {\rm{\beta}} \,{x_{\rm error}}

where α and β are factors that scale the contribution of each term.

The definition of the objective function (15) does not include constraints. The constraints are considered as the lower }{}{{\bf q}_{{1_l}}} and upper }{}{{\bf q}_{{1_u}}} joint limits for manipulator 1. Similarly, }{}{{\bf q}_{{2_l}}} and }{}{{\bf q}_{{2_u}}} define the lower and upper joint limits for manipulator 2, respectively. Let define the lower and upper joint limits for the cooperative system as }{}{{\bf x}_l} = {\left[ {\matrix{ {{\bf q}_{{1_l}}^T} & {{\bf q}_{{2_l}}^T} \cr } } \right]^T} and }{}{{\bf x}_u} = {\left[ {\matrix{ {{\bf q}_{{1_u}}^T} & {{\bf q}_{{2_u}}^T} \cr } } \right]^T}, with }{}{{\bf x}_l},{{\bf x}_u} \in {{\rm {\open R}}^{{n_1} + {n_2}}}. A joint value *x*_*j*_ is considered feasible if *xl*_*j*_ < *x*_*j*_ < *xu*_*j*_ for each joint *j* = 1,2,3,…,*n*_1_ + *n*_2_. To penalize unfeasible joint values, it is defined two penalty functions. The penalty function defined in Eq. (16) handles the constraints when *x*_*j*_ < *x*_*j*_. On the other hand, the penalty function given in Eq. (17) handles the constraints when *x*_*j*_ < *xu*_*j*_.

(16) }{}g({x_j}) = \left\{ {\matrix{ 0 {{\rm \bf if}\;\;{x_{{l_j}}} \lt {x_j}} \cr {{{\left( {{x_j} - {x_{{l_j}}}} \right)}^2}} {\rm \bf otherwise} \cr } } \right.

(17) }{}h({x_j}) = \left\{ {\matrix{ 0 {{\rm \bf if}\;\;{x_j} \lt {x_{{u_j}}}} \cr {{{\left( {{x_j} - {x_{{u_j}}}} \right)}^2}} {\rm \bf otherwise} \cr } } \right.

Finally, the two defined penalty functions are included into Eq. (15) to obtain the proposed objective function *f*′ that handles joint limits constraints. It is defined as

(18) }{}{f^{'}} = f + \sum\limits_{j = 1}^{{n_1} + {n_2}} {\rm \delta} \left[ {g\left( {{x_j}} \right) + h\left( {{x_j}} \right)} \right]

where δ scales the penalization term that is usually selected as a large constant. We recommend to use δ = 1,000. In conclusion, the optimal joint variable }{}{\bf x} is computed by solving the constrained optimization problem defined as

\matrix{ {\mathop {\min }\limits_{\bf x} {f^{\prime}}\left( {\bf x} \right),\,\,\text{subject\;to}\;{{\bf x}_{\bf l}} < {\bf x} < {{\bf x}_{\bf u}}} \cr }

Coordinates manipulators are physically interacting with each other to perform a common task. In this work, the relative motion between two mobile manipulators must be adequately computed to perform the task. The proposed objective function considers the relative position between the end-effector position of the two mobile manipulators. The relative constraints between the two mobile manipulators are not considered in this work.

### Self-adaptive differential evolution

The classical Differential Evolution (DE) is a population-based optimization algorithm (Storn & Price, 1997). The population is composed of individuals that represent potential solutions for a given global optimization problem. The DE algorithm performs three principal operations: mutation, crossover and selection, as a mechanism to improve the individuals during an iterative process called generations.

To begin, every individual }{}{\bf x}_i^G \in {{\rm {\open R}}^D} is generated randomly, where *i* = 1,2,3,…, *N* with *D* as the dimension of the problem, *N* as the total numbers of population members, and *G* is the current generation. The mutant operation consists in the computation of a mutant vector }{}{{\bf v}_i} as follows

(19) }{}{\bf v}_i^{G + 1} = {\bf x}_{{r_1}}^G + F\left( {{\bf x}_{{r_2}}^G - {\bf x}_{{r_3}}^G} \right)

where }{}F \in \left[ {0,2} \right] is the amplification factor, and three individuals are chosen randomly }{}{\bf x}_{{r_1}}^G, }{}{\bf x}_{{r_2}}^G and }{}{\bf x}_{{r_3}}^G with }{}{r_1},{r_2},{r_3} \in \left\{ {1,N} \right\} and *i* ≠ *r*_1_ ≠ *r*_2_ ≠ *r*_3_.

In the crossover operation, a trial vector }{}{\bf u}_i^{G + 1} is generated based on the following crossover scheme

(20) }{}u_{ji}^{G + 1} = \left\{ {\matrix{ {v_{ji}^{G + 1}} {{\bf if}\; {r_j} \le {C_R}\; {\bf or}\;j = {r_i}} \cr\hskip -6pt {{x_{ji}^G}} \hskip 5pt{{\bf if}\;{r_j} \gt {C_R}\; {\bf and}\; j \ne {r_i}} \cr } } \right.

where *C*_*R*_ ∈ [0,1] is the crossover constant with *j* = 1,2,3,…,*D*. The values *r*_*j*_ and *r*_*i*_ are random numbers computed as *r*_*j*_ ∈ [0,1] and }{}{r_i} \in \left\{ {1,D} \right\}. The value *r*_*i*_ ensures that the trial vector }{}{\bf u}_i^{G + 1} gets at least one element of the mutant vector }{}{\bf v}_i^{G + 1}.

In the selection operation, the trial vector }{}{\bf u}_i^{G + 1} is compared to the actual vector }{}{\bf x}_i^G with respect to the evaluation of an objective function *f*. This operation is performed as

(21) }{}{\bf x}_i^{G + 1} = \left\{ {\matrix{ {{\bf u}_i^{G + 1}} {{\bf if}\; f\left( {{\bf u}_i^{G + 1}} \right) \lt f\left( {{\bf x}_i^G} \right)} \cr\hskip -.5pc {{\bf x}_i^G} \hskip -3.2pc{\bf otherwise} \cr } } \right.

where, if }{}f\left( {{\bf u}_i^{G + 1}} \right) yields a better solution than }{}f\left( {{\bf x}_i^G} \right), then }{}{\bf u}_i^{G + 1} is considered as }{}{\bf x}_i^{G + 1}. On the other hand, }{}{\bf x}_i^G is retained to the next generation.

A new variant of DE is proposed in Fan, Xie & Zhou (2019). This variant is called self-adaptive mutation differential evolution, which includes a modified version to create the mutant vector }{}{\bf v}_i^{G + 1}. This mutation operation is given by

(22) }{}{\bf v}_i^{G + 1} = \left\{ {\matrix{ \hskip -3.7pc{{\bf x}_{{r_1}}^G + F\left( {{\bf x}_{{r_2}}^G - {\bf x}_{{r_3}}^G} \right)} {{\bf if}\; rand \lt 1 - {{\left( {G/{G_{\max}}} \right)}^2}} \cr {{\bf x}_b^G + F\left( {{\bf x}_{{r_1}}^G - {\bf x}_{{r_2}}^G + {\bf x}_{{r_3}}^G - {\bf x}_{{r_4}}^G} \right)} \hskip -5pc{\bf otherwise} \cr } } \right.

where }{}{\bf x}_b^G represents the population member with the best position, }{}{\bf x}_{{r_4}}^G is chosen randomly with }{}{r_4} \in \left\{ {1,N} \right\} and *i* ≠ *r*_1_ ≠ *r*_2_ ≠ *r*_3_ ≠ *r*_4_. Moreover, *G*_max_ indicates the total number of generations and *rand* ∈ [0,1]. The mutation operation based on }{}{\bf x}_{{r_1}}^G maintains the diversity of population for global exploration. The mutation based on }{}{\bf x}_b^G provides a mutation with local exploitation for a convergence accuracy and fast converge rate.

This new variant of DE proves to be superior than the classical DE algorithm. The self-adaptive mutation differential evolution is called SAMDECO by the authors (Fan, Xie & Zhou, 2019). In this work, SAMDECO is considered to be named as SDE for brevity.

### Inverse kinematics for cooperative mobile manipulators

This section introduces an inverse kinematics algorithm for cooperative mobile manipulator based on the SDE algorithm. Moreover, the inverse kinematics algorithm is considered to solve cooperative path tracking tasks.

The aim of the inverse kinematic is to compute the optimal joint configuration }{}{\bf x} for the whole cooperative system, given the desired end-effector position }{}{\bf t}_{{e_1}}^* and the relative position }{}{{\bf t}_r}. This relative position is used to compute the desired end-effector position }{}{\bf t}_{{e_2}}^*, Eq. (11). Every individual in SDE represents a set of joint configuration }{}{{\bf x}_i} \in {{\rm {\open R}}^D} for the whole cooperative system, where *D* = *n*_1_ + *n*_2_ indicates the total DOF of the system. The initialization of each individual can be computed as

(23) }{}{x_{ji}} = {x_{lj}} + ({x_{uj}} - {x_{lj}})\;r

where *r* ∈ [0,1] is a random number, *i* = 1,2,…,*N* and *j* = 1,2,…,*D*. The joint limits }{}{{\bf x}_l} and }{}{{\bf x}_u} must be provided since the inverse kinematics is considered as a constrained problem. The SDE algorithm was not designed to work with constrained optimization. Initially, the lower and upper joint limits are used to initialize the population as feasible joint solutions. However, the initialization does not guarantee that the SDE algorithm will converge to a feasible solution. Joint limits must be considered during the optimization process to provide a valid joint solution.

The forward kinematics can be used to compute the matrix transformation }{}^w{{\bf T}_{{e_1}}}\left( {{{\bf q}_1}} \right) and }{}^w{{\bf T}_{{e_2}}}\left( {{{\bf q}_2}} \right) with Eqs. (9) and (10), for each individual }{}{{\bf x}_i}. These matrices contain the position vectors }{}{{\bf t}_{{e_1}}} and }{}{{\bf t}_{{e_2}}} required to compute the position errors Eqs. (12) and (13). The vector }{}{{\bf x}_0} is chosen as an initial joint configuration. In a path tracking task, this vector becomes the previous joint variable needed to minimize the joint motion, Eq. (14). At this point, the objective function can be evaluated using Eq. (18). The parameter setting for the objective function α and β needs to be provided by the user. It is recommended to use α = 1.5 and γ = 0.6. This setting was determined by experimentation.

The SDE algorithm performs the mutation, crossover and selection operations to improve the individuals in each generation. The algorithm for the inverse kinematics of cooperative mobile manipulator based on SDE is given in Algorithm 1. The stop criteria is met when the SDE algorithm reaches the total number of generations or when the evaluation of the fitness function reaches an allowed tolerance *s*_tol_. The tolerance occurs when }{}s_{\rm stop} = t_{e_1}+ t_{e_2}.

**Algorithm 1:** Inverse kinematics for cooperative mobile manipulator based on SDE.

1: *f*′ ←objective function defined in Eq. (18)
2: set *N*, *F* ∈ [0,2] and *C_R_* ∈ [0,1] values
3: **for** each individual *i* **do**
4: initialize individual position **x***_i_* using Eq. (23)
5: **end**
6: **repeat**
7: **for** each individual *i* **do**
8: randomly choose }{}{\bf x}_{r1}^{G}, }{}{\bf x}_{r2}^{G} and }{}{\bf x}_{r3}^{G}
9: compute a mutant vector }{}{\bf v}_i^{G+1} using Eq. (22)
10: calculate a trial vector }{}{\bf u}_i^{G+1} using Eq. (20)
11: select the individual for next generation }{}{\bf x}_i^{G+1} using Eq. (21)
12: **until** stop criteria met
13: report results

**Figure 4:**
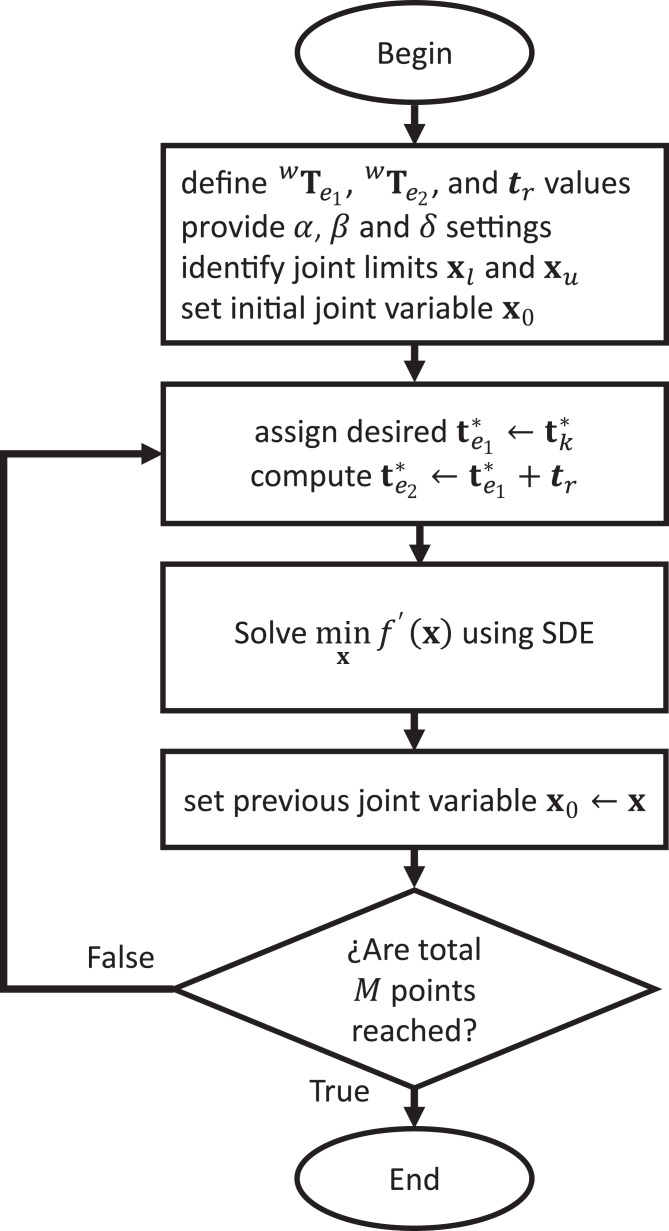
Cooperative path tracking algorithm.

Respect to the cooperative path tracking tasks, the considered path is divided into *k* = 1,2,3,…,*M* points, with *M* as the total number of points in the path. Then, the inverse kinematics for each point }{}{\bf p}_k^* is solved with Algorithm 1. The description of the cooperative path tracking algorithm is given in Fig. 4.

## Experimental results

The aim of the experiments is to test the proposed approach for solving cooperative path tracking tasks. A cooperative system with two mobile manipulators was considered for experiments. Moreover, a comparative study is included to compare the performance of CFPSO, FPA, SDE and KABC for the solution of the given path tracking tasks.

The considered mobile manipulator is based on the KUKA Youbot (López-Franco et al., 2018). It is conformed by a manipulator of five DOF, and an omnidirectional mobile platform of three DOF. With respect to the mobile manipulator kinematics, the transformation ^*w*^**T**_*b*_ can be computed with the mobile platform pose, which is given by *x*_*b*_, *y*_*b*_ and θ_*b*_. The constant transformation ^*b*^**T**_*m*_ is considered to be

}{}^b{{\bf T}_m} = \left[ {\matrix{ 1 0 0 {0.140} \cr 0 1 0 0 \cr 0 0 1 {0.151} \cr 0 0 0 1 \cr } } \right]

these values were obtained based on the KUKA Youbot technical specifications.

**Figure 5:**
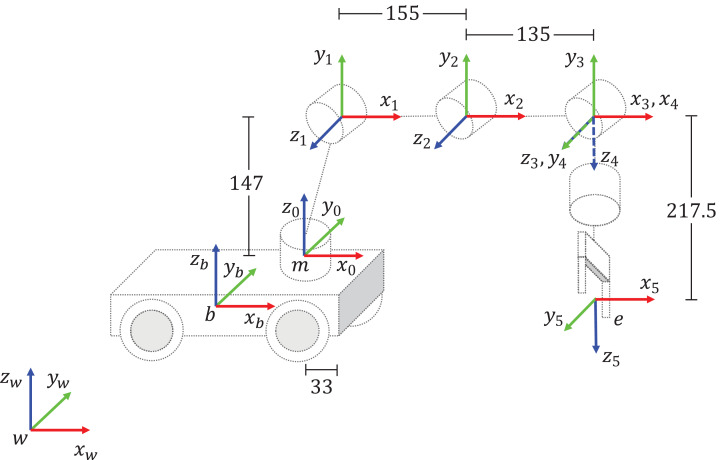
DH coordinate frame assignment for the KUKA Youbot. All measurements are given in millimeters (mm).

The DH table and the coordinate frame assignment for the KUKA Youbot are given in Table 1 and Fig. 5, respectively.

**Table 1:** DH table for KUKA Youbot manipulator.

Joint	*a* (mm)	α (rad)	*d* (mm)	θ (rad)
1	33	π/2	147	θ_1_
2	155	0	0	θ_2_
3	135	0	0	θ_3_
4	0	π/2	0	θ_4_
5	0	0	217.5	θ_5_

The DH table is used to compute the transformation ^*m*^**T**_*e*_. Moreover, the next homogeneous matrices represent the manipulator kinematics model

}{}^0{{\bf T}_1}({{\rm{\theta}} _1}) = \left[ {\matrix{ {\cos \left( {{{\rm{\theta}} _1}} \right)} 0 {\sin \left( {{{\rm{\theta}} _1}} \right)} {0.033\cos \left( {{{\rm{\theta}} _1}} \right)} \cr {\sin \left( {{{\rm{\theta}} _1}} \right)} 0 { - \cos \left( {{{\rm{\theta}} _1}} \right)} {0.033\sin \left( {{{\rm{\theta}} _1}} \right)} \cr 0 1 0 {0.147} \cr 0 0 0 1 \cr } } \right]

}{}^1{{\bf T}_2}({{\rm{\theta}} _2}) = \left[ {\matrix{ {\cos \left( {{{\rm{\theta}} _2}} \right)} { - \sin \left( {{{\rm{\theta}} _2}} \right)} 0 {0.155\cos \left( {{{\rm{\theta}} _2}} \right)} \cr {\sin \left( {{{\rm{\theta}} _2}} \right)} {\cos \left( {{{\rm{\theta}} _2}} \right)} 0 {0.155\sin \left( {{{\rm{\theta}} _2}} \right)} \cr 0 0 1 0 \cr 0 0 0 1 \cr } } \right]

}{}^2{{\bf T}_3}({{\rm{\theta}} _3}) = \left[ {\matrix{ {\cos \left( {{{\rm{\theta}} _3}} \right)} { - \sin \left( {{{\rm{\theta}} _3}} \right)} 0 {0.135\cos \left( {{{\rm{\theta}} _3}} \right)} \cr {\sin \left( {{{\rm{\theta}} _3}} \right)} {\cos \left( {{{\rm{\theta}} _3}} \right)} 0 {0.135\sin \left( {{{\rm{\theta}} _3}} \right)} \cr 0 0 1 0 \cr 0 0 0 1 \cr } } \right]

}{}^3{{\bf T}_4}({{\rm{\theta}} _4}) = \left[ {\matrix{ {\cos \left( {{{\rm{\theta}} _4}} \right)} 0 {\sin \left( {{{\rm{\theta}} _4}} \right)} 0 \cr {\sin \left( {{{\rm{\theta}} _4}} \right)} 0 { - \cos \left( {{{\rm{\theta}} _4}} \right)} 0 \cr 0 1 0 0 \cr 0 0 0 1 \cr } } \right]

}{}^4{{\bf T}_5}({{\rm{\theta}} _5}) = \left[ {\matrix{ {\cos \left( {{{\rm{\theta}} _5}} \right)} & { - \sin \left( {{{\rm{\theta}} _5}} \right)} & 0 & 0 \cr {\sin \left( {{{\rm{\theta}} _5}} \right)} & {\cos \left( {{{\rm{\theta}} _5}} \right)} & 0 & 0 \cr 0 & 0 & 1 & {0.2175} \cr 0 & 0 & 0 & 1 \cr } } \right]

where the transformation ^*m*^**T**_*e*_ is computed as

(24) }{}^m{{\bf T}_e} = {\,^0}{{\bf T}_1}({{\rm{\theta}} _1}){\,^1}{{\bf T}_2}({{\rm{\theta}} _2}){\,^2}{{\bf T}_3}({{\rm{\theta}} _3}){\,^3}{{\bf T}_4}({{\rm{\theta}} _4}){\,^4}{{\bf T}_5}({{\rm{\theta}} _5})

The joint variable }{}{\bf q} for the mobile manipulator is

}{}{\bf q} = {\left[ {\matrix{ {{x_b}} {{y_b}} {{{\rm{\theta}} _b}} {{{\rm{\theta}} _1}} {{{\rm{\theta}} _2}} {{{\rm{\theta}} _3}} {{{\rm{\theta}} _4}} {{{\rm{\theta}} _5}} \cr } } \right]^T}

where the joint values θ_1_−θ_5_ represent the joint configuration of the manipulator. Then, it is proposed to use }{}{{\bf q}_1} and }{}{{\bf q}_2} to represent the joint variable for mobile manipulator 1 and 2, respectively. The joint configuration for the whole cooperative system is given by }{}{\bf x} = {\left[ {\matrix{ {{\bf q}_1^T} & {{\bf q}_2^T} \cr } } \right]^T}.

The joint limits were obtained based on the KUKA Youbot technical specifications. The lower joint limits for the mobile manipulator 1 (}{}{{\bf q}_{{1_l}}}) and mobile manipulator 2 (}{}{{\bf q}_{{1_l}}}) are set to

}{}{{\bf q}_{{1_l}}} = {{\bf q}_{{2_l}}} = {\left[ {- 1.5\; - 1.5\; - 3.1416\; - 2.9496\;- 1.1345\; - 2.6180\; - 1.7837\; - 2.9234} \right]^T}

where the lower joint limits for the cooperative system is }{}{{\bf x}_l} = {\left[ {\matrix{ {{\bf q}_{{1_l}}^T} & {{\bf q}_{{2_l}}^T} \cr } } \right]^T}. Similarly, the upper joint limits for the mobile manipulator 1 (}{}{{\bf q}_{{1_u}}}) and mobile manipulator 2 (}{}{{\bf q}_{{1_u}}}) are set to

}{}{{\bf q}_{{1_u}}} = {{\bf q}_{{2_u}}} = {\left[ {1.5\quad 1.5\quad 3.1416\quad 2.9496\quad 1.5708\quad 2.5482\quad 1.7890\quad 2.9234} \right]^T}

where the upper joint limits for the cooperative system is }{}{{\bf x}_u} = {\left[ {\matrix{ {{\bf q}_{{1_u}}^T} & {{\bf q}_{{2_u}}^T} \cr } } \right]^T}.

Four trajectories with different degrees of difficulty are considered for testing the proposed approach. Each trajectory is divided into *k* = 1,2,3,…,*M* points, where *M* is the total number of points in the path. The vector }{}{\bf t}_k^* = {\left[ {\matrix{ {{x_k}} & {{y_k}} & {{z_k}} \cr } } \right]^T} defines the *k*th trajectory point, and it is used as a target for the cooperative inverse kinematics. The considered trajectories are provided below

\matrix{ {} {\bf Trajectory\; 1: Sinusoidal} \cr {{x_k}}\;{ = 0.5} \cr {{z_k}}\;{ = 0.4 + 0.05\sin \left( {30\,{y_k}} \right)} \cr {{y_k} \in \left[ {0.5,1.5} \right]}}

\matrix{ {\bf Trajectory\; 2: Circular} \cr {{x_k}}\;{ = 0.5} \cr {{y_k}}\;{ = 0.05\cos \left( {{{\rm{\theta}} _k}} \right)} \cr {{z_k}}\;{ = 0.4 + 0.05\sin \left( {{{\rm{\theta}} _k}} \right)} \cr {{{\rm{\theta}} _k} \in \left[ {0,2{\rm \pi} } \right]}}

\matrix{ {\bf Trajectory\; 3: Trapezoidal} \cr {{x_k}}\;{ = 0.5} \cr {{r_k}}\;{ = 0.4 + 0.1\sin \left( {30\,{y_k}} \right)} \cr {{y_k} \in \left[ {0.5,1.5} \right]} \cr {{z_k}}\;{ = \left\{ {\matrix{ {0.45} \; {{\bf if}\;{r_k} \gt 0.45} \cr {0.35} \; {{\bf if}\;{r_k} \lt 0.35} \cr {{r_k}} \; {\bf otherwise} \cr } } \right.} \cr }

\matrix{ {\bf Trajectory\; 4: Rosecurve} \cr {{x_k}}\;{ = 0.5} \cr {{y_k}}\;{ = {r_k}\cos \left( {{{\rm{\theta}} _k}} \right)} \cr {{z_k}}\;{ = 0.4 + {r_k}\sin \left( {{{\rm{\theta}} _k}} \right)} \cr {{r_k}}\;{ = 0.035 + 0.015\cos \left( {3\,{{\rm{\theta}} _k}} \right)} \cr {{{\rm{\theta}} _k} \in \left[ {0,2{\rm\pi} } \right]} \cr }

where a total of *M* = 200 points were selected. Moreover, the relative vector for the cooperative tasks is set to }{}{{\bf t}_r} = {\left[ {\matrix{ 0 { - 1} 0 \cr } } \right]^T}, and the initial joint configuration is defined as

}{}{{\bf x}_0} = {\left[ {0\;\;0.5\;\;{\rm\pi} /4\;\;0\;\;{\rm \pi} /2\;\;-{\rm \pi} /4\;\;\pi /4\;\;0\;\;0\;\;-0.5\;\;{\rm \pi} /4\;\;0\;\;{\rm \pi} /2\;\;-{\rm \pi} /4\;\;{\rm \pi} /4\;\;0} \right]^T}

The parameter setting for the considered meta-heuristics algorithms is conducted as follows: The particular parameter setting of CFPSO are the cognitive factor *C*_1_ = 2.05, the social factor *C*_2_ = 2.05, and ϕ = 4.1 for the constriction factor, see Collinsm & Shen (2017). The settings for FPA are the switch probability *P* = 0.8 for local and global pollination and a step size λ = 1.5 for strength of the pollination, see recommendation in Yang (2012). In the SDE algorithm, it was considered to use the amplification factor *F* = 0.5 and crossover constant *C*_*R*_ = 0.8 based on Fan, Xie & Zhou (2019). The particular parameter setting for KABC are the forager bees population *P*_*f*_ = 20, the onlooker bees population *P*_*o*_ = *N* − *P*_*f*_, and stagnation limit parameter *L* = (*N* * *D*)/2, see Karaboga & Basturk (2007) and El-Sherbiny, Elhosseini & Haikal (2018). With respect to the common parameter settings, it was considered to use a population size of *N* = 30 individuals, a total of *G*_max_ = 1,000 generations, and a tolerance of *s*_stop_ = 1 × 10^−4^.

The simulations are presented in two parts. In the first part, the comparative study is presented. To test the performances of the considered meta-heuristics algorithms, the inverse kinematics results for each point *k* in the path were used to compare the statistical variations. The position error }{}t_{1_{\rm error}}+ t_{2_{\rm error}} and the motion error *x*_error_ are reported. Additionally, the execution time in seconds is also reported, where the specifications of the test machine are Intel Core i7-4770 CPU 3.4 GHz and 16.0 GB of RAM. To display graphically the statistical variation for the results, box plots were used. The best algorithms will show the smaller data distribution, the lower values results, and the less quantities of outliers. In the second part, it is considered to run multiple experiments on every trajectory to verify the robustness of the SDE algorithm because the mobile manipulator is redundant and there is more than one set of inverse solutions.

**Figure 6:**
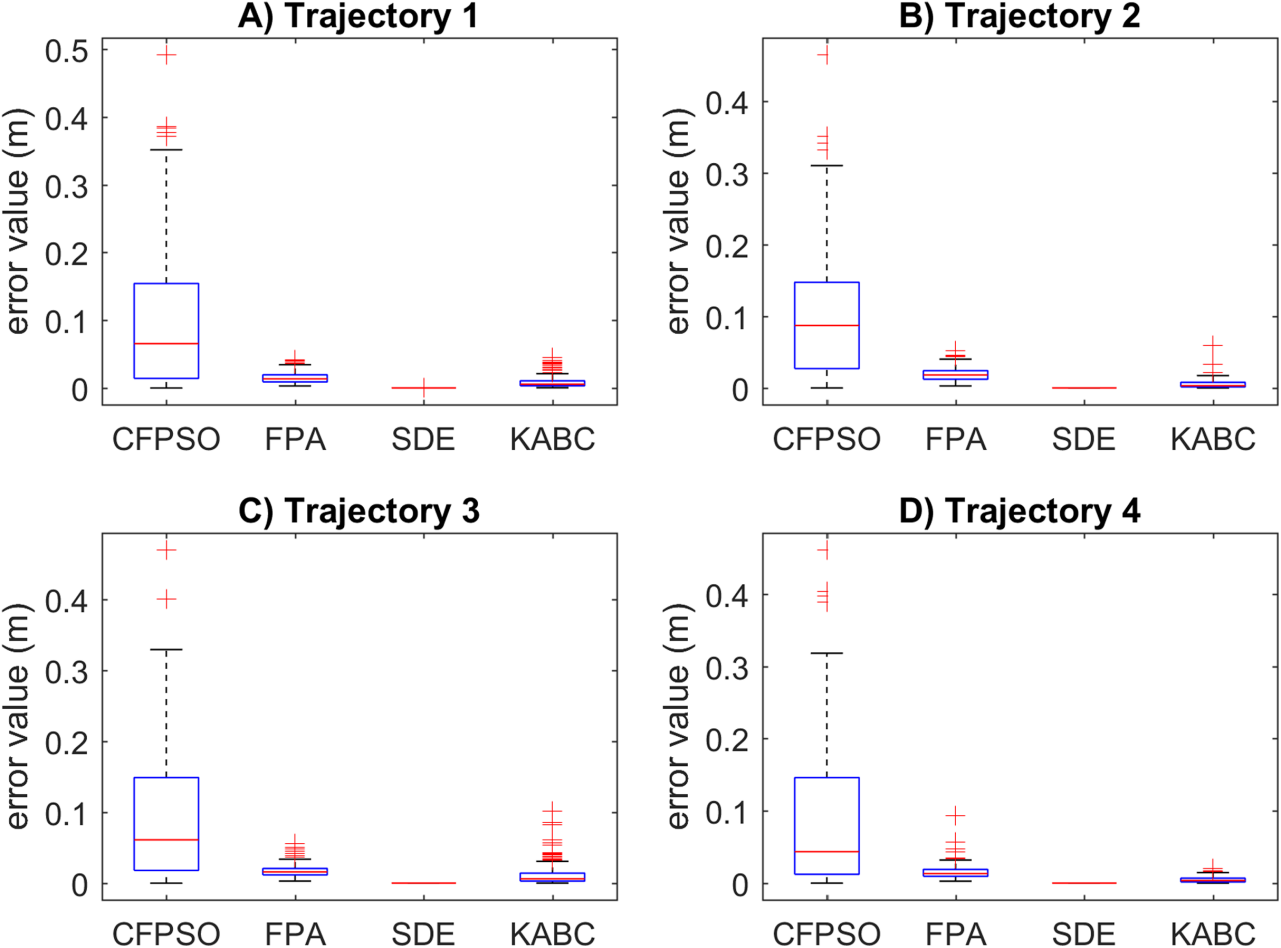
Position error results for the cooperative path tracking tasks. (A–D) Simulation results for the Sinusoidal, Circular, Trapezoidal, and Rosecurve trajectories, respectively.

The results of the first part of the simulations are shown below. The position error results for the cooperative path tracking are presented in Fig. 6. The CFPSO algorithm reports the largest data distribution in all trajectories. In contrast, the SDE algorithm shows the smallest data distribution with the lowest value results. The FPA and KABC algorithms have similar data distribution, with better value results than CFPSO. However, KABC performs better than FPA with lower value results. In general, the SDE algorithm presents more consistent results with precision. On the other hand, the CFPSO, FPA and KABC algorithms present outliers results and lower precision.

**Figure 7:**
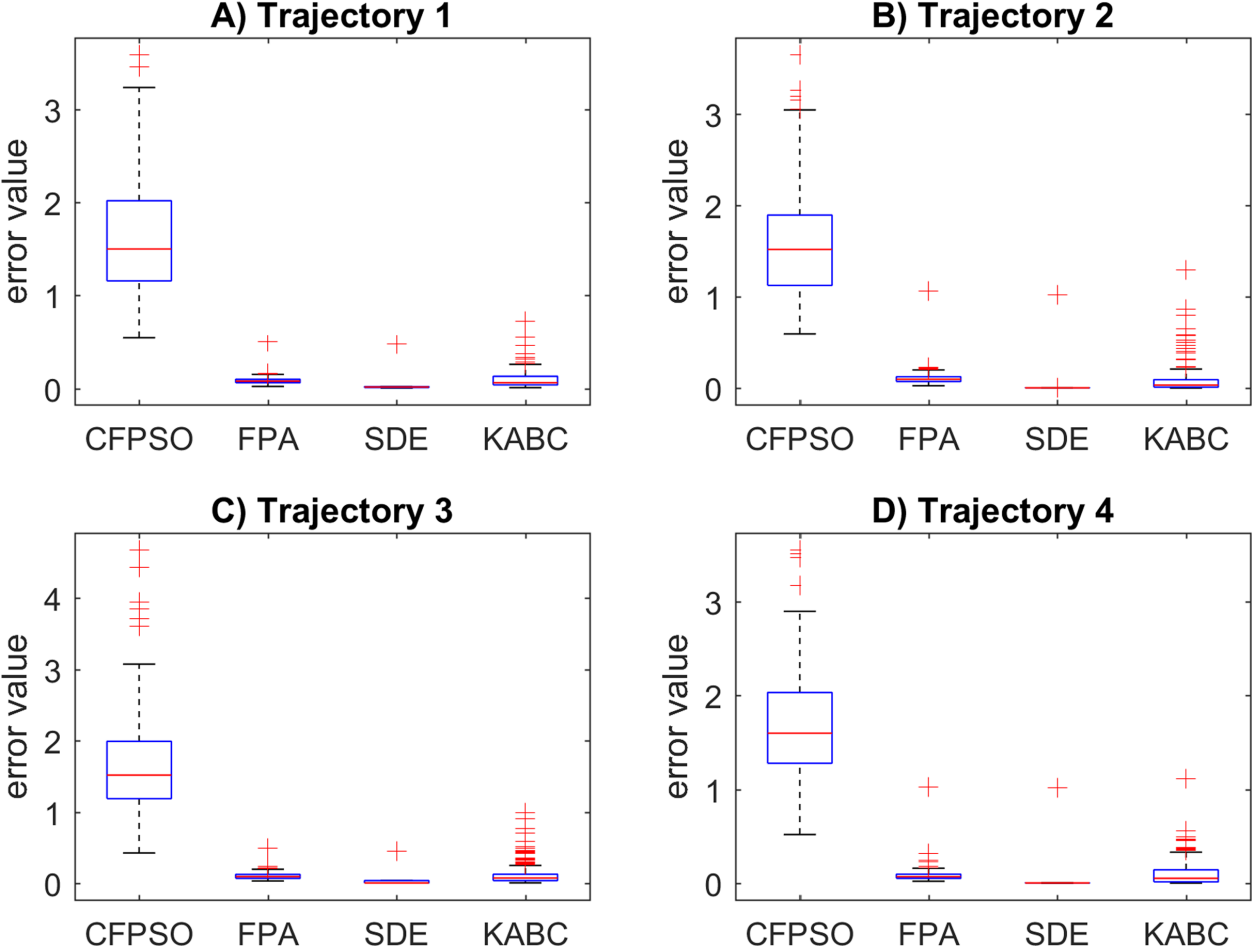
Motion error results for the cooperative path tracking tasks. (A–D) Simulation results for the Sinusoidal, Circular, Trapezoidal, and Rosecurve trajectories, respectively.

The motion error results for the cooperative path tracking are illustrated in Fig. 7. It is crucial to mention that the more motion error, the more joint changes are presented in the path following. A big motion error produces abrupt changes in the joint motion. The SDE algorithm reported smaller data distribution with slower motion errors. These results are precise and a mean error value of 0.1033 is obtained, in general. In contrast, the CFPSO algorithm presents the larger data distribution with a mean motion error value of 1.6296. This is not a reliable result due to the presence of abrupt changes in the joint motion. The FPA and KABC performed similarly, with mean error values of 0.0959 and 0.0142, respectively. Finally, the CFPSO and KABC contain more quantity of outliers than the other algorithms.

**Figure 8:**
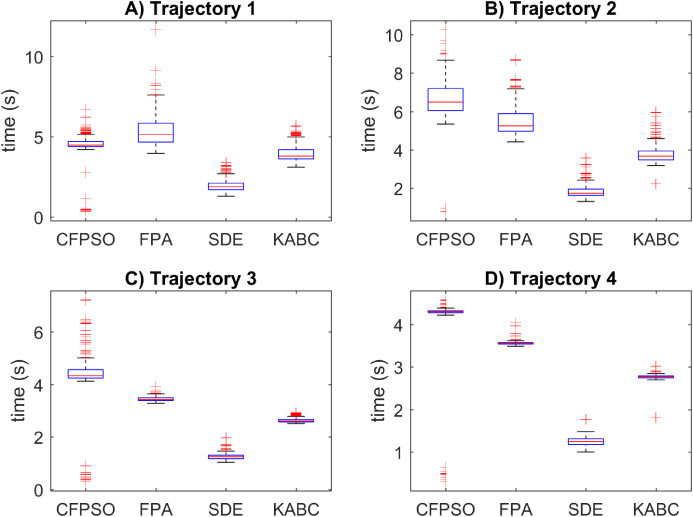
Execution time results for the cooperative path tracking tasks. (A–D) Simulation results for the Sinusoidal, Circular, Trapezoidal, and Rosecurve trajectories, respectively.

The execution time results for the cooperative path tracking are reported in Fig. 8. The SDE algorithm outperformed the other with the smaller execution time results with a mean value of 1.5828 s in general. The CFPSO and FPA algorithms have similar time results, with mean values of 4.9043 s and 4.4768 s. However, the KABC algorithm performed better than CFPSO and FPA, with a mean value of 3.2938 s. The position error results in Fig. 6, suggests that most of the inverse kinematics results did not reach the allowed tolerance, and CFPSO, FPA and KABC reached the total number of iterations. For this reason, the execution time is bigger than SDE. In this case, all compared algorithms have similar data distribution, and the reported data shows outliers, which indicates that execution time is not precise.

Additionally, Table 2 shows the root mean square (RMS) results for the cooperative path tracking tasks. The table contains the position error results for both mobile manipulator position errors }{}t_{1_{\rm error}} and }{}t_{2_{\rm error}}. The SDE algorithm outperformed the others with better accuracy RMS results, with values below to 1 × 10^−4^ which is the tolerance allowed. The RMS values of the other algorithm did not reach the tolerance. The KABC performed better than FPA with lower RMS values, and CFPSO obtained the worst RMS results.

**Table 2:** RMS values for the position error }{}t_{i_{\rm error}} results of mobile manipulator *i*. The best results are highlighted in bold.

Trajectory		CFPSO	FPA	SDE	KABC
1	*}{}t_{1_{\rm error}}* (m)	4.9927 × 10^−2^	7.6444 × 10^− 3^	**6.8059** × 10^−5^	5.2494 × 10^−3^
	*}{}t_{2_{\rm error}}* (m)	4.8456 × 10^−2^	7.3751 × 10^−3^	**6.8210** × 10^−5^	3.5065 × 10^−3^
2	}{}t_{1_{\rm error}} (m)	4.6660 × 10^−2^	9.2997 × 10^−3^	**6.3131** × 10^−5^	3.2665 × 10^−3^
	*}{}t_{2_{\rm error}}* (m)	5.4808 × 10^−2^	9.5702 × 10^−3^	**6.3632** × 10^−5^	2.1096 × 10^−3^
3	*}{}t_{1_{\rm error}}* (m)	4.9342 × 10^−2^	8.2215 × 10^−3^	**6.7407** × 10^−5^	3.9660 × 10^−3^
	*}{}t_{2_{\rm error}}* (m)	4.3967 × 10^−2^	8.9793 × 10^−3^	**6.4637** × 10^−5^	7.6678 × 10^−3^
4	*}{}t_{1_{\rm error}}* (m)	4.8983 × 10^−2^	7.5944 × 10^−3^	**6.6192** × 10^−5^	2.5669 × 10^−3^
	*}{}t_{2_{\rm error}}* (m)	3.9981 × 10^−2^	8.0709 × 10^−3^	**6.5240** × 10^−5^	2.0876 × 10^−3^

**Figure 9:**
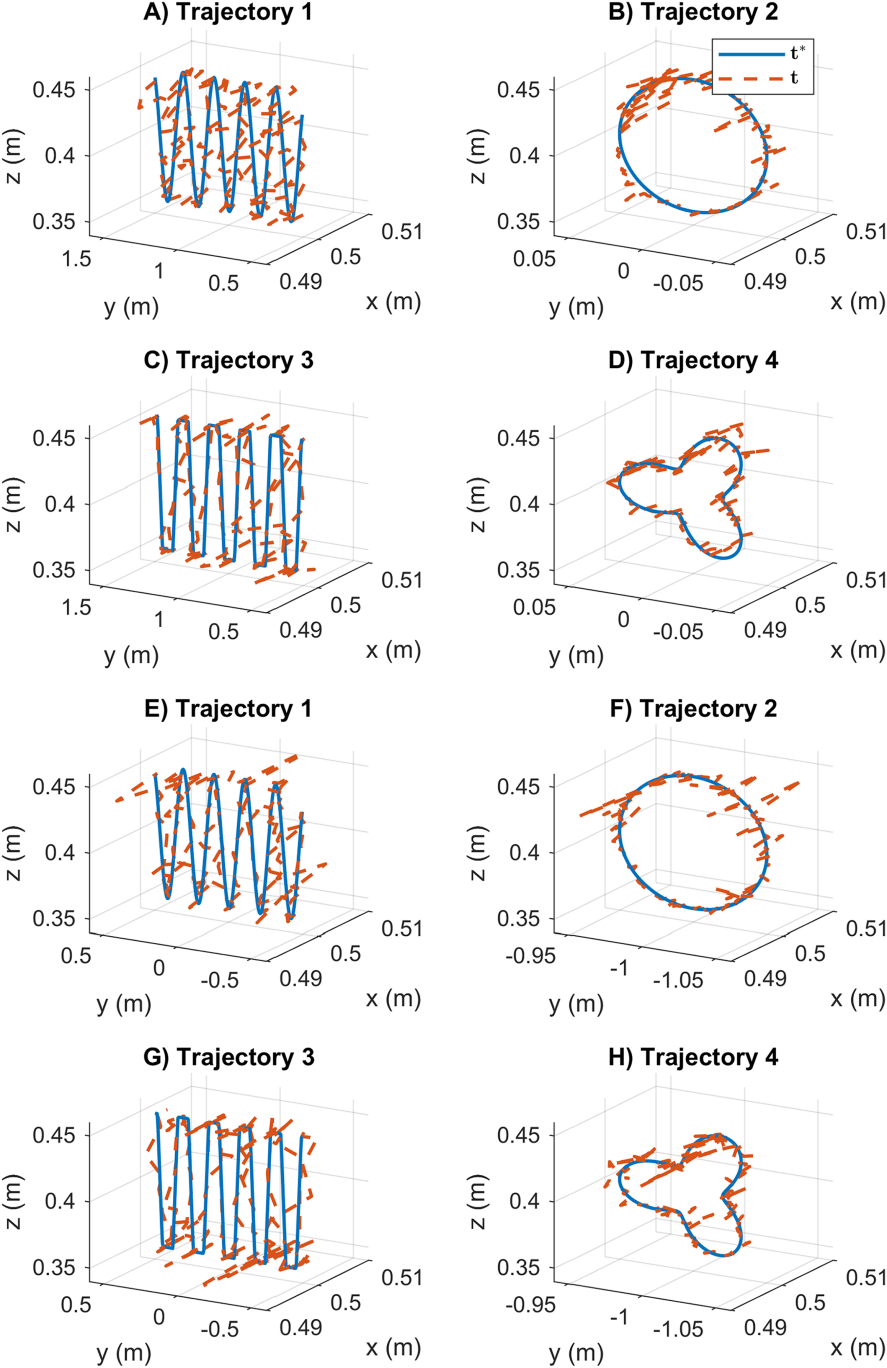
Path tracking results for the cooperative tasks based on the KABC algorithm. The vector t* represents the desired end-effector position, and vector t is the obtained position. (A–D) The results for mobile manipulator 1. (E–H) The results for mobile manipulator 2.

Based on the results given so far, the SDE and KABC performed better than the other algorithms. For this reason, the path tracking and joint motion results are displayed graphically for comparison. First, the path tracking results for the cooperative tasks using the KABC algorithm are presented in Fig. 9. The path tracking results reported are not consistent, and the position error results have low precision. The statistical variation of position error during the path tracking is indicated with large data distribution, see Fig. 6.

**Figure 10:**
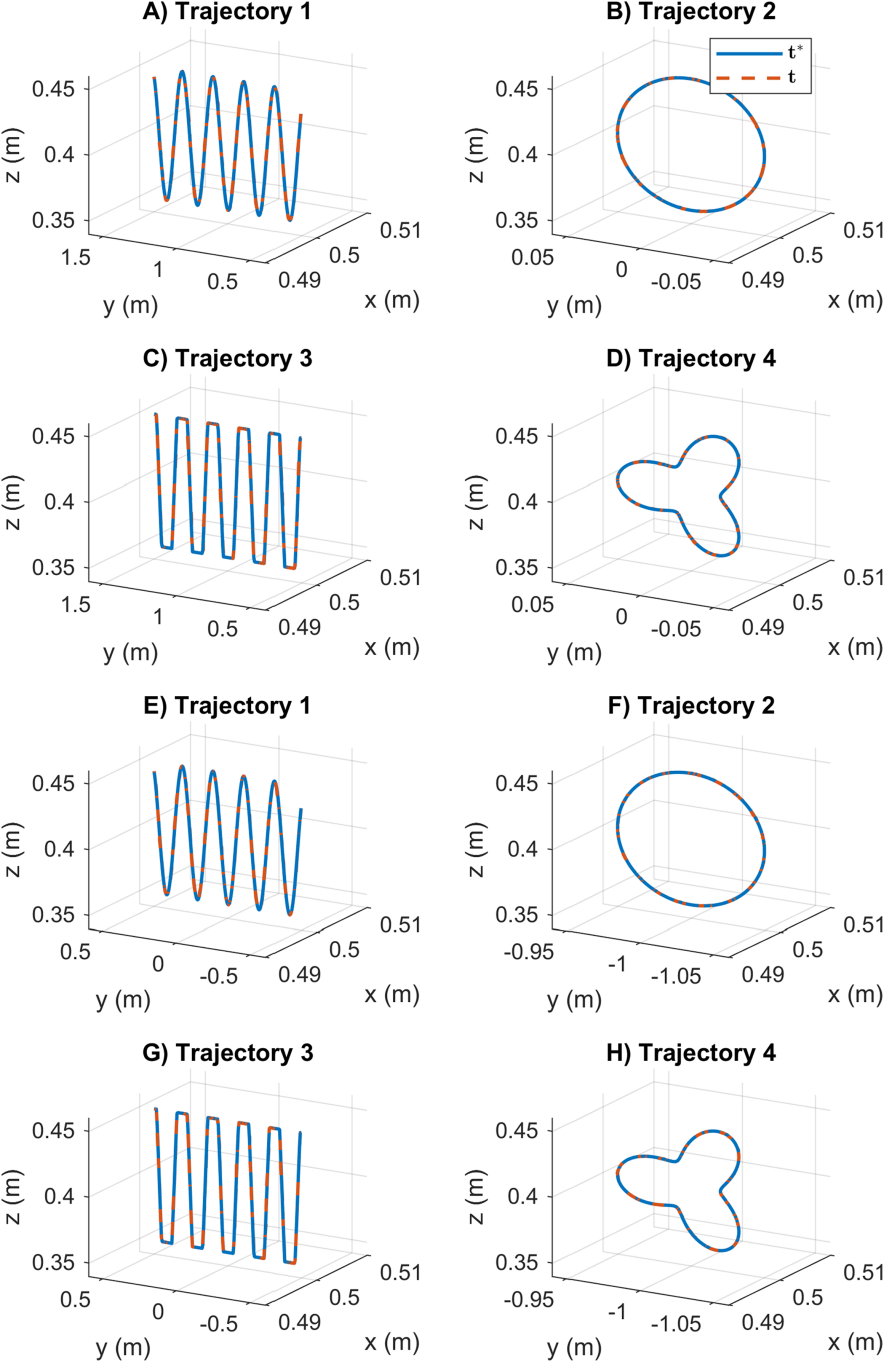
Path tracking results for the cooperative tasks based on the SDE algorithm. The legend t* represents the desired end-effector position, and t is the obtained position. (A–D) The results for mobile manipulator 1. (E–H) The results for mobile manipulator 2.

Moreover, the Fig. 10 reports the path tracking results using the SDE algorithm. In this case, the reported results indicate that SDE has precise and accurate path tracking values. The desired end-effector position of manipulator *i* and the obtained position are considered identically. The statistical variation of position error during the path tracking is represented by a small data distribution and low value results, see Fig. 6. These results indicate that SDE outperformed the KABC algorithm concerning better path tracking results.

**Figure 11:**
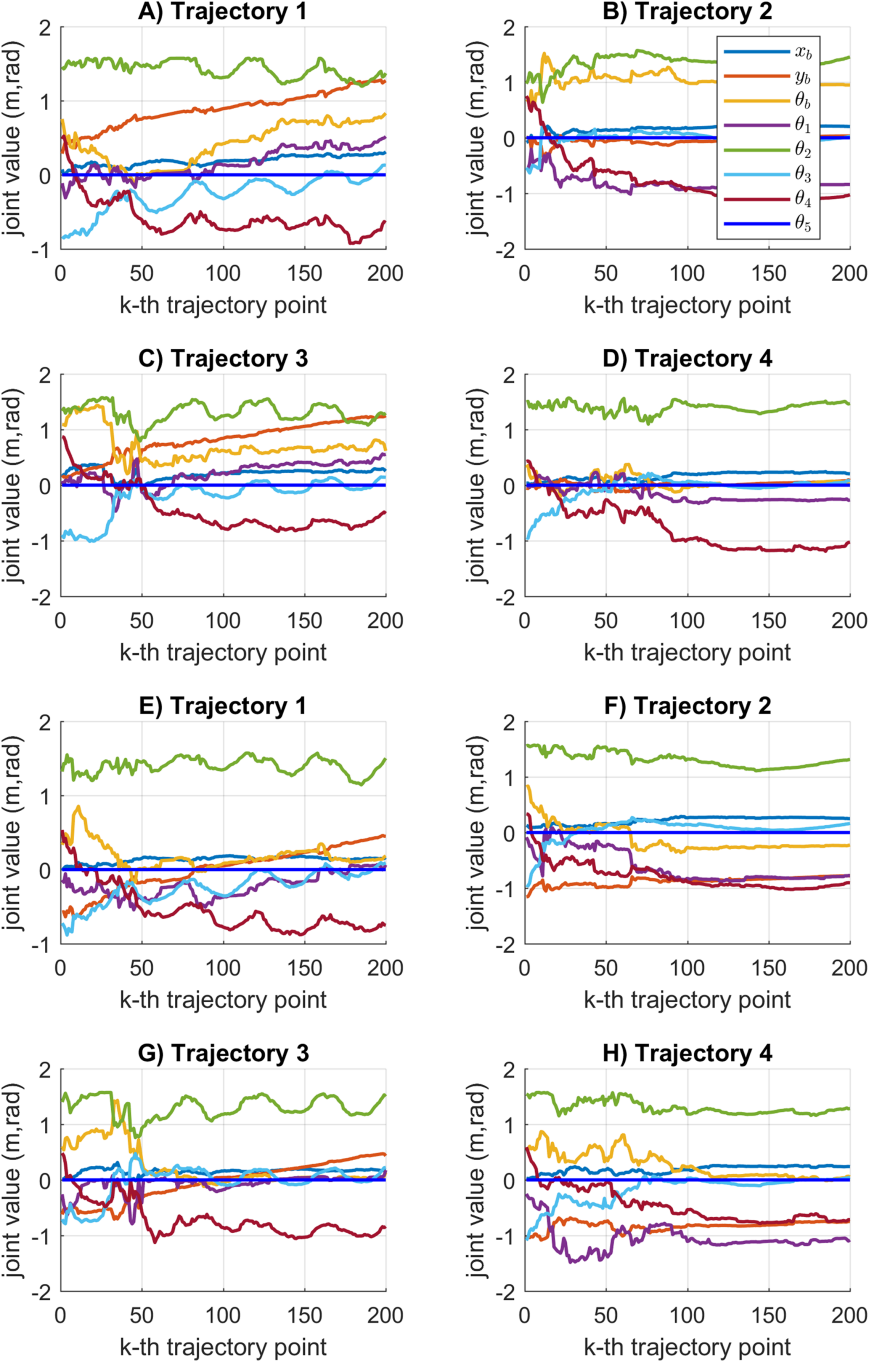
Joint motion results based on the KABC algorithm. (A–D) The results for mobile manipulator 1. (E–H) The results for mobile manipulator 2.

The joint motion results obtained by the KABC algorithm are illustrated in Fig. 11. In this case, the joint values during the cooperative tasks present a rough motion. The statistical variation of the motion error during the path tracking indicates large data distribution with big value results see Fig. 7. It is important to notice that rough motion may cause hardware failure.

**Figure 12:**
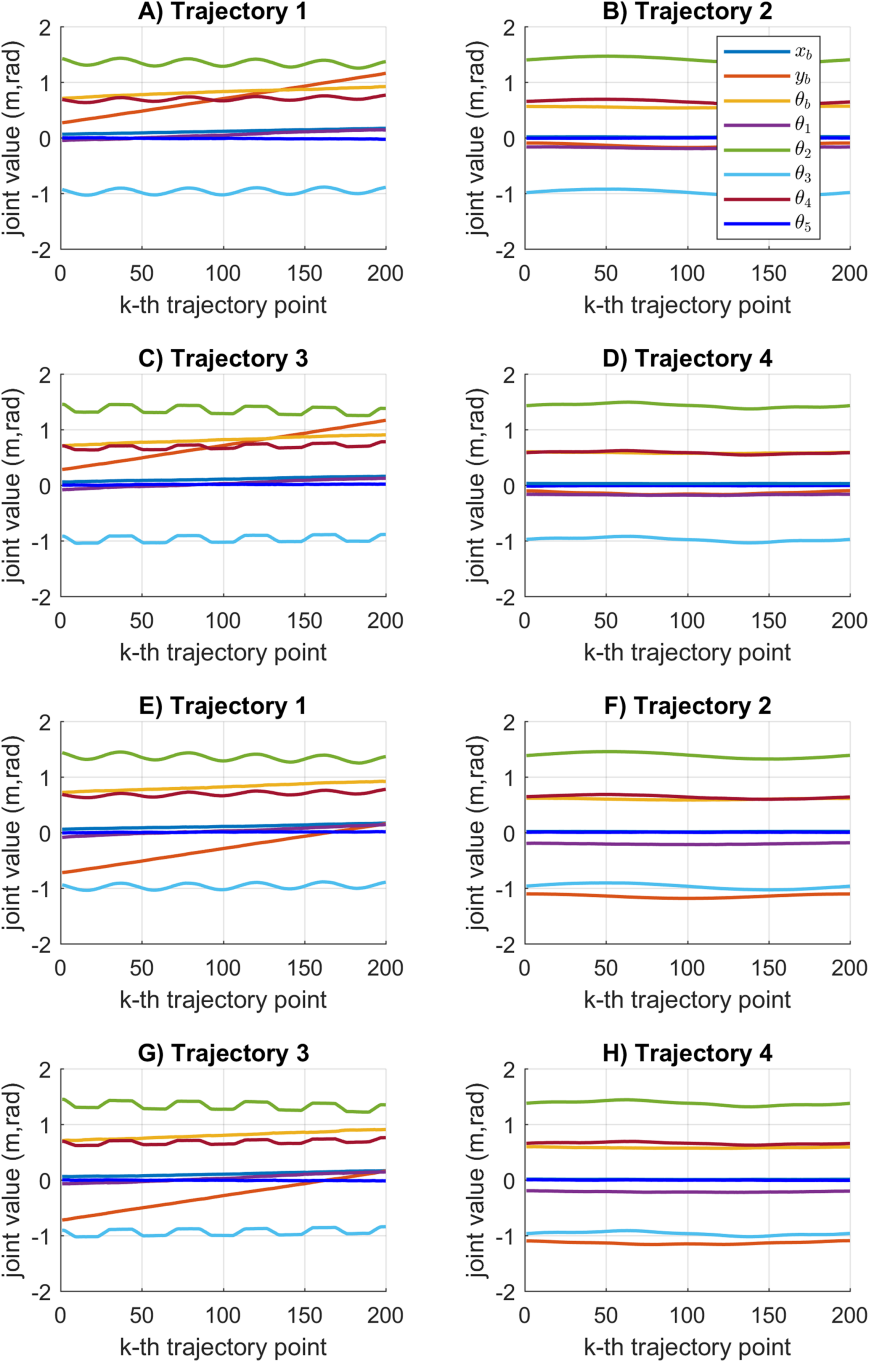
Joint motion results based on the SDE algorithm. (A–D) The results for mobile manipulator 1. (E–H) The results for mobile manipulator 2.

Figure 12 shows the joint motion results obtained by the SDE algorithm. In this case, the joint values obtained during the cooperative task reports smooth motion results. The statistical variation of the motion error during the path tracking is represented by a small data distribution with low value results see Fig. 7. The SDE algorithm obtains reliable motion results compared to the KABC algorithm.

Considering the results reported in this experimental section, the SDE algorithm outperformed the CFPSO, FPA and KABC algorithms, in general. The SDE algorithms reported better position and motion error results with a lower execution time. Additionally, path tracking and motion results validate the precision and accuracy values provided by SDE. The CFPSO algorithms performed poorly in all tests, and the FPA and KABC algorithms performed similarly in general. The SDE algorithm proves to be more adequate to solve the inverse kinematics for cooperative mobile manipulators than the compared algorithms.

The results of the second part of the simulations are shown below. In this case, the initial joint is defined as

}{}{{\bf x}_0} = {\left[ {0.3\;\;0.5\;\;{\rm \pi} /4\;\;0\;\;{\rm \pi} /2\;\;0\;\;{\rm \pi} /2\;\;{\rm \pi} /4\;\;0.3\;\;- 0.5\;\;- {\rm \pi} /4\;\;0\;\;{\rm \pi} /2\;\;0\;\; {\rm \pi} /2\;\;- {\rm \pi} /4} \right]^T}

Moreover, the SDE algorithm runs 25 times, and the set of joint solutions are shown graphically. The rest of the experimental setup is the same as the previous experiments.

**Figure 13:**
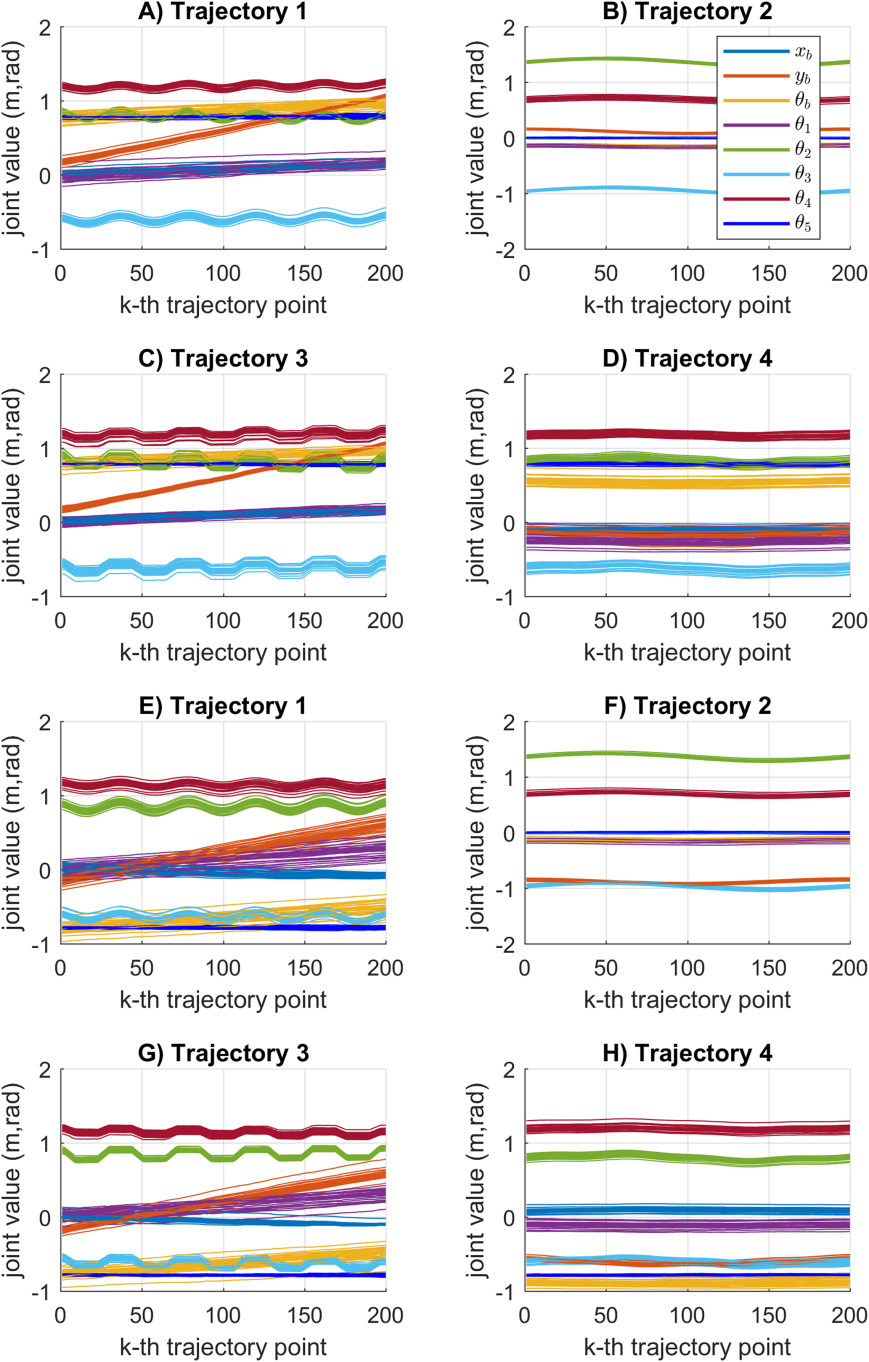
Multiples joint motion results based on the SDE algorithm. (A–D) The results for mobile manipulator 1. (E–H) The results for mobile manipulator 2.

Figure 13 shows the results of the multiple joint motion results for every considered trajectory. As can be seen, multiples joint motion results are achieved. The joint motion results are very similar because the proposed objective function considers the minimum error between the current and the previous joint configuration. Moreover, the joint solutions for the first point on the trajectory will depend on the initial joint values }{}{{\bf x}_0}. If a different set of joint solutions is required, then the initial joint values must be different.

One of the advantages of the SDE algorithm is its mutation mechanism that provides a balance between the exploration and exploitation characteristics. This improves the performance of SDE with respect to convergence accuracy and fast converge rate. The experimental results indicate that thanks to these characteristics, the SDE algorithm has the lowest execution time, and the lowest position and displacement errors. In addition, the statistical analysis demonstrates the consistency of the results obtained with the smallest data distribution. The SDE algorithm also shows that it can reach the allowed tolerance while the other compared algorithms are not. All these results guarantee the convergence to the global optimum value and the computation of smooth trajectories.

## Conclusions

This work introduced an approach to solving the inverse kinematics for cooperative mobile manipulators based on self-adaptive differential evolution (SDE). Simulation experiments were performed to prove the effectiveness of the proposed approach for solving cooperative path tracking tasks. It was considered to include four trajectories with different degrees of difficulty. The simulations also included a comparison among CFPSO, FPA, SDE, and KABC algorithms. The inverse kinematics results were used to compare the algorithms statistically. The SDE algorithm outperformed the other with consistent statistical results, such as the smallest data distribution and the lowest value results. The execution time of SDE also is the best, with a mean value of 1.5828 seconds. Moreover, the path tracking errors of SDE reported precise and accurate results, with RMS position error results below 0.1 mm. Additionally, the motion error results of SDE reported smooth joint displacement during path tracking. The FPA and KABC algorithms performed similarly. They reported position error results near 1 mm. However, their statistical results with respect to the motion error results present large data distribution. This inconvenience is observed with rough joint displacements. The CFPSO performed poorly in general, its position error result is greater than 10 mm, and motion error results indicate abrupt changes in the joint displacement. The CFPSO algorithm is not recommended to solve the inverse kinematics for cooperative mobile manipulators. The experimental setup considered two mobile manipulators based on the KUKA Youbot system, which is conformed by an omnidirectional mobile platform with three DOF, and a manipulator with five DOF. However, the proposed approach can be used to solve the inverse kinematics for cooperative omnidirectional mobile manipulators with *n* DOF. As future research, the proposed approach can be extended to solve cooperative dual-arm systems attached to the same mobile platform. It is also appealing to include cooperative mobile manipulators with nonholonomic mobile platforms.

## Additional Information and Declarations

### Data Availability

The following information was supplied regarding data availability: Data and code are available at GitHub: https://github.com/JesusHernandezBarragan/PeerJ---Inverse-kinematics-for-cooperative-mobile-manipulators-based-on-self-adaptive-differential-e.git.
